# Case report: Novel deletions in the 6p21.33 involving the *CSNK2B* gene in patients with Poirier-Bienvenu neurodevelopmental syndrome and literature review

**DOI:** 10.3389/fmed.2024.1441573

**Published:** 2024-10-18

**Authors:** Xuan Zhang, Hongjuan Lu, Yichen Ji, Wei Sun

**Affiliations:** Department of Neurology, Xuanwu Hospital, Capital Medical University, Beijing, China

**Keywords:** 6p21.33 deletion, CNSK2B, POBINDS, epilepsy, biphasic patterns, digital anomalies

## Abstract

**Background:**

Seizures have been identified in most patients with *CSNK2B*-related Poirer-Bienvenu Neurodevelopment syndrome (POBINDS). Detailed descriptions of seizure phenotypes, various genotypes, and long-term follow-up visits are required for clinicians to provide reasonable clinical management for such patients.

**Case summary:**

We report two new Chinese patients with varying sizes of 6p21.33 deletions encompassing the *CSNK2B* gene who presented with intellectual disability and seizures. Furthermore, we conducted a literature review of previously reported patients with 6p21.33 deletions or *CSNK2B* variants. We summarized and analyzed the clinical characteristics of these patients with seizures. The occurrence of a biphasic pattern of epilepsy and pharmacoresistant epilepsy in patients with *CSNK2B* variants is severely underestimated. One of our patients underwent a long follow-up period and presented with comprehensive disease progression.

**Conclusion:**

Our data suggest that the *CSNK2B* variant or 6p21.33 deletion should be considered in patients with intellectual disability and epilepsy, especially those characterized by biphasic patterns and digital anomalies.

## Introduction

In 2017, Poirier et al. (1) first identified de novo mutations of the *CSNK2B* gene in two patients with intellectual disability with or without seizures who were later diagnosed with Poirier-Bienvenu neurodevelopmental syndrome (POBINDS) (1). CK 2 beta (*CSNK2B*), located in chromosome 6p21.33, encodes the β subunit of the CK2 protein complex (2). CK2 comprises two catalytic subunits (α and/or α’) and two regulatory subunits (β) and is ubiquitously expressed in various cells, especially in the brain. The β subunit of CK2 plays roles in recognition and anchoring, ensuring and promoting the phosphorylation reaction (2). CK2 is a serine-threonine protein kinase that phosphorylates hundreds of physiological substrates in human tissue and participates in all important cellular processes, including cell proliferation, differentiation, apoptosis, synaptic transmission, DNA replication, and repair (2, 3).

Poirier-Bienvenu neurodevelopmental syndrome is characterized by developmental delay, intellectual disability, epilepsy, and facial abnormalities. More than 70 cases have been reported, most of which are in the infancy or early stages of disease (4, 5). Genotype-phenotype correlations and comprehensive descriptions of the course of the disease are required. Here, we present two patients harboring varying-sized deletions in 6p21.33, where the *CSNK2B* gene is located. Further, we offer a comprehensive literature review on detailed seizure descriptions in patients with *CSNK2B* gene variants, aiming to assist in achieving early diagnosis and intervention.

## Cases presentation

**FIGURE 1:**
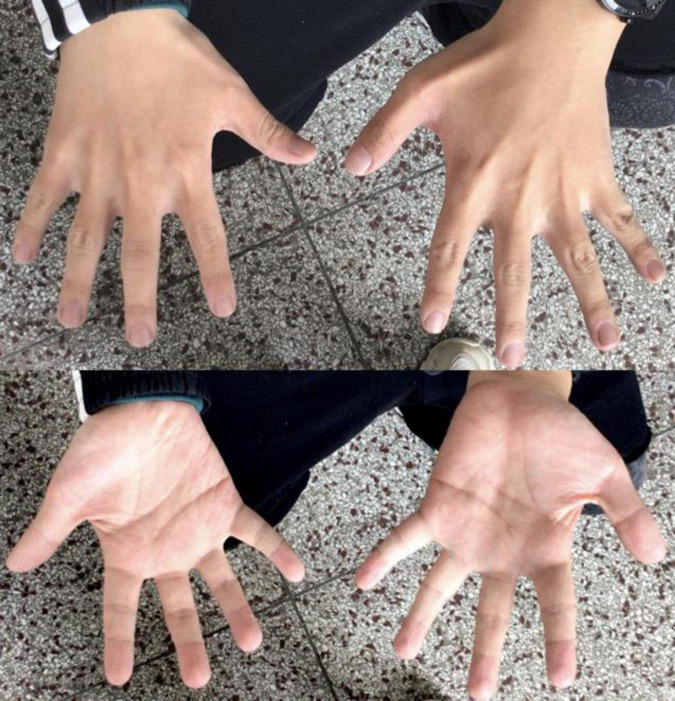
Digital anomalies of patient 1.

**FIGURE 2:**
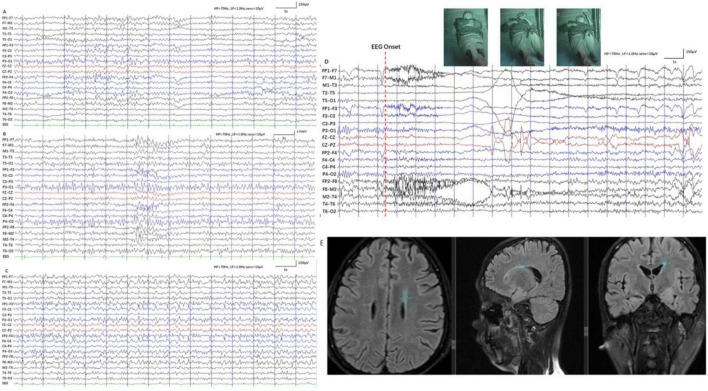
EEG and Imaging data of patient 1. **(A–C)** EEG of patient 1 at 20 years old, 23 years old and 24 years old. Background of EEG slows down at 20 years old **(A)**, paroxysmal slow waves lasting for less than 3 s at 23 years old **(B)**, lasting for 20–30 s at 24 years old **(C)**. Progressive slowing of the electroencephalographic (EEG) background is prominent. **(D)** EEG of patient 1 at 24 years old. The ictal EEG was characterized by right cerebral focus with diffused electrodecremental event, subsequently evolving into bilateral hemispheres. **(E)** MRI images of patient 1 at 23 years. MRI: revealed abnormal signal in right-sided centrum semiovale.

Patient 1 was a 24-year-old Chinese male, the second child of healthy, non-consanguineous parents, with a healthy older sister. After an uneventful pregnancy and delivery, the mother gave birth at 28. The patient’s weight, height, and head circumference were normal at birth. The developmental milestone of this patient was within the normal range before the age of three. The patient’s family history was unremarkable. The first seizure occurred at the age of three years, triggered by fever; after that, he experienced febrile seizures approximately two to three times a year. The patient developed afebrile seizures at 16 years of age. The seizures were characterized by sudden episodes of blinking and twitching of the unilateral corners of the mouth, followed by ipsilateral limb twitching that lasted approximately 10 min. He presented with seizures in clusters, with a frequency of more than ten episodes per month. At the age of 18, seizures were controlled with valproate (VPA), lamotrigine (LTG), and oxcarbazepine (OXC) treatment. After a seizure-free period of 1 year, the seizures recurred, and the patient was resistant to antiepileptic medications (AED). A biphasic pattern of epilepsy was prominent in this patient and was characterized by an easy response to AEDs, followed by recurrent refractory seizures in adolescence. After the onset of epilepsy, the patients’ cognitive and language skills gradually decreased. Physical evaluation revealed dysmorphic features, including large low-set ears, a wide-base nose, a prominent forehead, a protuberant eyebrow arch, a high anterior hairline, thin hair, a broad left thumb, clinodactyly of the left fifth digit, and bilateral brachydactyly (Figure 1). The progressive slowing of the electroencephalography (EEG) in the background is prominent. The interictal EEG showed multifocal spikes and spike waves sporadically at an early age and presented continuous spikes and waves during sleep with age. Diffused electrodecremental events were observed over the right frontotemporal region at the beginning of the clinical seizures, and then 5–6 Hz theta activity was recruited over the bilateral anterior regions with interruption of motor artifacts (Figure 2). Brain magnetic resonance imaging (MRI) revealed bilateral temporal horn enlargement and abnormal signals in the right central semiovale (Figure 2).

**FIGURE 3:**
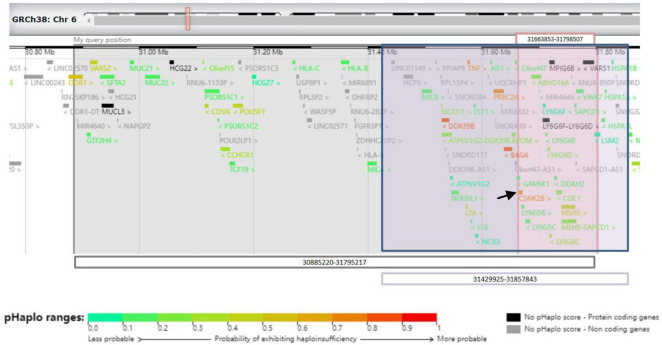
Schematic representation of 6p21.33 deletions in three cases from the UCSC Genome Browser. Gray-shaded area shows the area of 6p21.33 deletion in patient 1. Pink-shaded area shows the area of 6p21.33 deletions in patient 2. Purple-shaded area shows the area of 6p21.33 deletion in patient of Ikuko et al. The black arrow shows CSNK2B gene.

Next-generation sequencing was performed for the patient and his parents. A size of 0.91 Mbp heterozygous deletion of the short arm of chromosome 6 [(Chr6: 30885220-31795217) × 1] was revealed, deleting the OMIM-listed gene in Figure 3. Neither parent harbored the same deletion as the patient. The deletion regions encompassed 85 genes, and haploinsufficiency of *CSNK2B* could explain the clinical phenotype of our patient.

Patient 2 was a 22-month-old Chinese male with non-consanguineous parents and a healthy elder brother. The patient was born at term after an uneventful pregnancy and delivery. The global development was slightly delayed; he lifted his head at the age of 4 months and sat unassisted at the age of 8 months. The patient cannot walk independently and only speaks “baba” or “mama.” At five months, he experienced episodes of seizures characterized by tonic unilateral extremities lasting for approximately 2–3 s, with a frequency of several times a day. Seizures were controlled with VPA. EEG and MRI were normal. Facial features were not prominent due to her younger age.

Next-generation sequencing was performed for the patient and his parents. A size of 0.135 Mbp de novo heterozygous deletion in the short arm of chromosome 6 [(Chr6: 31663853-31798507) × 1] was observed in patient 2 (Figure 3). Neither parent harbored the same deletion as the patient. The deletion regions encompassed 22 genes, and the haploinsufficiency of *CSNK2B* could account for the clinical phenotype of our patient.

Approximately 91 cases have been reported in the literature; epilepsy was present in 83/91 of the patients (91%), usually before the age of 2 years. Drug-resistant epilepsy and a biphasic pattern of epilepsy account for 35% of patients with *CSNK2B*-related epilepsy. We compared the final follow-up age differences between patients with pharmacoresponsive epilepsy and those with pharmacoresistant epilepsy or biphasic epilepsy. The last follow-up age of patients with pharmacoresistant epilepsy or a biphasic pattern of epilepsy was significantly higher than that of patients with pharmacoresponsive epilepsy (*P* < 0.05). The main clinical, genetic, epilepsy, EEG, and brain MRI features of *CSNK2*B-related pharmacoresistant epilepsy and the biphasic pattern of epilepsy are summarized in Tables 1, 2.

**TABLE 1:** Summary of clinical features of patients with CSNK2B related biphasic pattern of epilepsy.

	Age	CSNK2B	Mutation protein	Onset age	Seizure type, frequency	Drug	Seizure-free period	Relapse age	Seizure type	Drug	Other neurologic features
Bonanni et al. (13)	14Y/M	c.27G > A	p. Trp9Ter	7M	GTCS, 2.5/D	VPA	5Y	8Y	Focal, 1/D	CBZ, VPA (10Y, seizure-free)	Mild ID, mild SI
Ernst et al. (5)	12Y/M	c.27del	p.Trp9Ter	4M	My	na	na	na	RSE	VPA, CZP, RUF, ZNS, PHT, PB, TPM, LCM, KD, VNS	Profound ID
	26Y/M	c.542del	p.Asn181fs	10M	GTCS, My, AA	na	na	na	RSE	na	Severe ID
	22Y/M	c.557++1G > A	na	2.5M	GTCS, My, A, T	na	na	na	RSE	na	Severe ID
Orsini et al. (14)	7Y/F	c.368–2A > G	na	10M	GTCS, 1/M	VPA	5Y	7Y	GTCS	VPA	Mild ID
Di Stazio et al. (4)	11Y/F	c.T116G	p.Leu39Arg	5Y	A,	ESM,VPA		7Y	F	VPA, LTG	Mild ID
Selvam et al. (15)	17Y/M	c.139 C > T	p. R47	11M	MA. Multiple per day	LTG,OXC, TPM	10Y	15Y	na	na	Mild ID
Nakashima et al. (16)	15Y/F	c.533_534insGT	p.Pro179Tyrfs49	2M	F,GTCS, daily	Clobazam nitrazepm ZNS,ESM, CZP,PHT, PB, ACTH	na	9Y	LGS, daily	AAZ, RUF, potassium bromide, TPM, LEV, LTG,	Profound ID
	7Y/M	c.494A > G	p.His165Arg	5D	F	LEV, PB	na	7Y	na	LEV, PB, TPM, CBZ	Profound ID
Our case1	24Y/M	6p21.33 deletions		3Y	F	VPA, LTG, OXC	1Y	19Y	F	LCS, VPA, CLB PER	Mild ID

DD, developmental disability; ID, intellectual disability; SI, speech impairment; My, myoclonic; RSE, refractory status epilepticus; AA, atypical absences; GTCS, generalized tonic clonic seizure; A, absences; T, tonic; ESM, Ethosuximide; MA, myoclonic-atonic seizures; LGS, Lennox-Gastaut syndrome; ZNS, zonisamide; CZP, clonazepam; PHT, phenytoin; PB, Phenobarbital; LEV, levetiracetam; LTG, lamotrigine; TPM, topiramate; ACTH, Adrenocorticotropic hormone; CLB, Clobazam; PER, Perampanel; Nitrazepam; RUF, rufinamide; acetazolamide, potassium bromide, AAZ, acetazolamide; na, not available.

**TABLE 2:** Summary of clinical features of patients with CSNK2B related pharmaco-resistant epilepsy.

	Age	CSNK2B	Mutation protein	Onset	Seizure type’ frequency	AEDs	EEG	ID
Poirier et al. (1)	19Y/M	c.175+2T > G	p.Val25Metfs13.	18M	My 1/10D	LTG, VPA, LEV, LB, ZNS	Generalized spike/(poly)spike waves discharges and a slow background	mild ID
Li et al. (17)	6Y/M	c.560 T > G	p.L187R	1Y	GTCS, FS, M, dozens/D	LEV, TPM, VPA, CZP	A Diffuse slow spike, multiple spike-slow and slow wave discharge	Profound ID
Di Stazio et al. (4)	27M/M	c.384_394del	p.Met132LeufsTer110	3M	FS, GTCS, SE, M	VPA, LEV, CZP	Diffuse paroxysmal abnormalities	mild ID
Wilke et al. (18)	7M/F	c.494A > G	p.His165Arg	20D	M, F	Pharmacoresistent	na	Profound ID
Zhang et al. (19)	30M/F	c.462_465del	p.Asp155Alafs70	4M	GTCS	LEV, VPA	Abnormal EEG	Profound ID
Asif et al. (8)	9Y/M	c.175+1G > C	na	3M	F, GTCS	LTG, VPA, CLB	Slow background,	Moderate ID
	21M/F	c.175+1G > C	na	1Y	T	LEV, PB	Normal	normal
Ernst et al. (5)	6Y/M	c.303C > A	p.Tyr101Ter	6m	MA	LTG, VPA, ZNS	A brief generalized discharge arising from a mildly slow background	Profound ID
	6Y/M	c.303C > A	p.Tyr101Ter	10m	MA	LTG, VPA	Frontally predominant generalized polyspikes	Profound ID
	12Y/M	c.27del	p.Trp9Ter	4m	M,RSE	VPA, CLN, RUF, ZNS, PHT, PB, TPM, LCM, KD, VNS	Generalized polyspikes and polyspike and wave at ∼2 Hz	Profound ID
	26Y/M	c.542del	p.Asn181fs	10m	GTC myoclonic, atypical absences; RSE	na	na	Severe ID
	21M/M	c.78_83dup	p.Glu27_Asp28dup	5m	absences, GTC	ZNS, VPA, LEV	na	Mild ID
	31Y/M	c.94G > A	p.Asp32Asn	2Y	absences, tonic-spasms	na	na	moderate ID
	8Y/F	c.139C > T	p.Arg47Ter	2Y	drop attacks, head drops with staring of eyes	ETX, CLB	na	mild ID
	11Y/F	c.558-2A > G	na	1–2 Y	focal onset, GTC, drop attacks	LEV, LTG	na	mild ID
	13Y/F	c.2T > A	p.Met1?	1 Y	na	KD, LEV	na	mild ID
	11Y/M	c.181G > T	p.Glu61Ter	2 m	GTC	PHT, LTG, FBM	na	mild ID
	22Y/M	c.557+1G > A	na	2.5 m	GTC, myoclonic, absences, tonic; RSE		na	severe ID
	12Y/M	c.94G > A	p.Asp32Asn	7 Y	A	VPA, ETX	na	moderate ID
Yang et al. (20)	3Y/M	c.494A > G	p.His165Arg	5 D	M	PB, TPM, LEV	More sharp waves in the central, apical, and midline areas	Profound ID
Orsini et al. (14)	1.3Y/F	c.332G > A	p.Arg111His	na	GTCS, infantile spasms	na	Focal and multifocal discharges with burst suppression	profound ID/DD
	6.5Y/F	c.116T > G	p.Leu39Arg	5 Y	CAE,F	ETX, ZNS, LTG	Multifocal epileptiform discharges	profound ID/DD

DD, developmental disability; ID, intellectual disability; SI, speech impairment; My, myoclonic; RSE, refractory status epilepticus; AA, atypical absences; GTCS, generalized tonic clonic seizure; A, absences; T, tonic; ESM, Ethosuximide; MA, myoclonic-atonic seizures; LGS, Lennox-Gastaut syndrome; ZNS, zonisamide; CZP, clonazepam; PHT, phenytoin; PB, Phenobarbital; LEV, levetiracetam; LTG, lamotrigine; TPM, topiramate; ACTH, Adrenocorticotropic hormone; Clobazam, Nitrazepam, rufinamide, acetazolamide, potassium bromide, VNS, vagal nerve stimulator ETX, ethosuximide, ZNS, zonisamide, CLB, Clobazam; na, not available.

## Literature review

A systematic literature search was performed using PubMed and the Web of Science. MeSH and title/abstract were used for all eligible studies that mainly focused on the *CSNK2B* mutation or 6p21.33 deletion. We included (1) publications written in English, (2) clinical case reports and series with sufficient clinical data, and (3) diagnoses confirmed by genetic testing. Data from all eligible studies was analyzed and discussed by two reviewers. We additionally reported our two patients.

We carefully read all of the articles to extract the clinical information for the patients and we included all the patients with *CSNK2B* mutation or 6p21.33 deletion described in the analysis. Approximately 91 cases have been reported in the literature; epilepsy was present in 83/91 of the patients (91%), usually before the age of 2 years. Drug-resistant epilepsy and a biphasic pattern of epilepsy account for 35% of patients with *CSNK2B*-related epilepsy. The last follow-up age of patients with pharmacoresistant epilepsy or a biphasic pattern of epilepsy was significantly higher than that of patients with pharmacoresponsive epilepsy (*P* < 0.05). The main clinical, genetic, epilepsy, EEG, and brain MRI features of *CSNK2*B-related pharmacoresistant epilepsy and the biphasic pattern of epilepsy are summarized in Tables 1, 2.

## Discussion

We present two patients with varying sizes of 6p21.33 deletions, including 0.135 Mbp and 0.91 Mbp, respectively, and provide a literature review of all the reported clinical cases on this subject. To date, only one case has been reported in the literature: Ikuko et al. reported a 7-year-old boy with a 0.43 Mbp deletion of 6p21.33 (6). A set of overlapping phenotypes with mild developmental delays, seizures, or febrile convulsions was observed in these three patients. According to the data from DECIPHER,^1^ the overlapping deleted regions encompassing 22 genes (Figure 3). Among them, *CSNK2B* showed high predictive scores for haploinsufficiency. *CSNK2B* gene was first identified by Poirier in patients with POBINDS (1). Thus, the haploinsufficiency of *CSNK2B* gene accounts for these clinical features. In addition, we observed more clinical phenotypes along with the evolution of the disease in Patient 1, suggesting a potentially progressive disease nature of *CSNK2B*-related POBINDS.

Despite the variable size of 6p21.33 deletions, all three patients shared an overlapping region (Figure 1) with a size of 0.135 Mb deletion. Terminal 4p deletion leads to Wolf–Hirschhorn syndrome (WHS), a contiguous gene deletion syndrome. The phenotypic severity of WHS is closely related to the size of the 4 p deletion (7). In contrast to WHS, all phenotypes presented by our patients could be explained by haploinsufficiency of the *CSNK2B* gene, which is located in an overlapping region (mentioned above). We speculated that the size of the 6p21.33 deletion does not correspond with the severity of the clinical phenotype.

Maria et al. reported that the c.94G > A variant of *CSNK2B* spawned a distinct phenotype characterized by cranial and digital anomalies, termed Intellectual Disability-Craniodigital Syndrome (IDCS), which is distinguishable from POBINDS (8). They suggested that haploinsufficiency of *CSNK2B* variants is the underlying pathomechanism of POBINDS. In contrast, a dominant-negative effect of *CSNK2B* variants contributes to the IDCS phenotype (8). In Patient 1, in addition to cranial anomalies and intellectual disability with seizures, digital anomalies were prominent, suggesting that both IDCS and POBINDS belong to a continuous spectrum of *CSNK2B*-associated phenotypes. The 6p21.33 deletion in this patient indicated that haploinsufficiency of *CSNK2B* had a predominant effect on the phenotype. The underlying pleiotropic effects of the variants may contribute to the variability of *CSNK2B*-associated phenotypes.

Up to date, approximately 91 patients with *CSNK2B* variants have been reported (5, 9–12). Epilepsy is a common symptom (approximately 91%). Epilepsy in patients with POBINDS begins early, usually before the age of 2 years, and generalized tonic or tonic-clonic seizures (GTCS) are the most common seizure type. Approximately 14.3% of patients had febrile convulsions or seizures. Drug-resistant epilepsy accounts for 37% (31/83) of patients with *CSNK2*B-related epilepsy. Similar to the observation of Ernst et al. (5) that generally seizure diminishes with age, but described 3 patients with worsening seizure frequency aged 7–12 years, we also find a biphasic pattern of epilepsy, which is characterized by an early age onset that is easily responsive to ASMs, followed by recurrent refractory seizures in adolescence, and was documented in 12% (10/83) of patients with epilepsy. We compared the final follow-up age differences between patients with pharmacoresponsive epilepsy and those with pharmacoresistant epilepsy or biphasic epilepsy. The last follow-up age of patients with pharmacoresistant epilepsy or a biphasic pattern of epilepsy was significantly higher than that of patients with pharmacoresponsive epilepsy. Although it has been reported that *CSNK2*B-related epilepsy outcomes are highly variable, we believe that the occurrence of a biphasic pattern of epilepsy and pharmacoresistant epilepsy in patients with *CSNK2B* variants is severely underestimated because the diagnosed age and last follow-up time of most patients are usually infants. Approximately 10 patients with a biphasic pattern of epilepsy were reported, and all of them had a relatively long follow-up duration, averaging 14.5 years (Tables 1, 2). The main clinical, genetic, epilepsy, EEG, and other neurological features are summarized in Table 1. Among these patients, we observed that seizure onset started in the first year of life in 80% of patients and within 5 years of age in 100%. During this phase, seizures were responsive to ASMs. After various periods of seizure freedom, ranging from 1 to 10 years, seizures recur in adolescence and tend to be pharmaco-resistant (Tables 1, 2). ID/DD was observed in 90% of the patients with *CNSK2B*-related epilepsy. Patients with pharmacoresistant epilepsy frequently showed more severe cognitive impairment, especially in situations in which seizures were not controlled for a long time (Tables 1, 2). Clinical management of patients with *CNSK2B*-related epilepsy requires aggressive treatment of seizures to avoid worsening neurodevelopmental outcomes.

In conclusion, our study identified two patients with rare 6p21.33 deletions encompassing the *CSNK2B* gene. Drawing from our observations, it seems that *CSNK2B* haploinsufficiency might play a decisive role in clinical manifestations. Our study further expands the phenotype-genotype spectrum of *CNSK2B*, thereby facilitating better the clinical management of this condition. Future researchs should focus on developing more effective therapies for *CNSK2B*-related epilepsy.

## Data Availability

The raw data supporting the conclusions of this article will be made available by the authors, without undue reservation.

## Footnotes

1
https://decipher.sanger.ac.uk
